# The first selective VAP-1 inhibitor in China, TT-01025-CL: safety, tolerability, pharmacokinetics, and pharmacodynamics of single- and multiple-ascending doses

**DOI:** 10.3389/fphar.2024.1327008

**Published:** 2024-04-29

**Authors:** Yuanxun Yang, Lei Yu, Zejuan Sheng, Hui Lin, Zuyi Weng, Wei Song, Bei Cao, Yu Zhao, Yingsheng Gao, Shumao Ni, Huimin Wang, Tingting Ma, Lei Huang, Caixia Sun, Juan Li

**Affiliations:** ^1^ Phase I Clinical Trials Unit, Nanjing Drum Tower Hospital, Affiliated Hospital of Medical School, Nanjing University, Nanjing, China; ^2^ TransThera Sciences (Nanjing), Inc., Nanjing, China

**Keywords:** TT-01025-CL, vascular adhesion protein-1 inhibitor, non-alcoholic fatty liver disease, non-alcoholic steatohepatitis, clinical study, pharmacokinetics, pharmacodynamics

## Abstract

**Introduction:** TT-01025-CL is an oral, irreversible small molecule that potently inhibits vascular adhesion protein-1 (VAP-1) for the treatment of inflammation associated with non-alcoholic steatohepatitis (NASH). The objectives of this study were to evaluate the safety/tolerability, pharmacokinetics, and pharmacodynamics of TT-01025-CL, a VAP-1 inhibitor, in healthy Chinese volunteers.

**Methods:** Double-blind, placebo-controlled, dose-escalation studies were conducted in subjects randomized to receive oral once-daily TT-01025-CL (ranges: 10–300 mg [single dose]; 20–100 mg for 7 days [multiple doses]) or placebo under fasting conditions. Safety and tolerability were monitored throughout the study. Pharmacokinetic (PK) parameters were determined using non-compartment analysis. The activity of semicarbazide-sensitive amine oxidase (SSAO)-specific amine oxidase and the accumulation of methylamine in plasma were evaluated as pharmacodynamic (PD) biomarkers.

**Results:** A total of 36 (single-dose group) and 24 (multiple-dose group) subjects were enrolled in the study. No serious adverse events (AEs) were reported, and no subject discontinued due to an AE. All treatment-emergent adverse events (TEAEs) were mild and moderate in intensity. No dose-dependent increase in the intensity or frequency of events was observed. TT-01025-CL was rapidly absorbed after administration. In the single-ascending dose (SAD) study, median T_max_ ranged from 0.5 to 2 h and mean t_1/2z_ ranged from 2.09 to 4.39 h. PK was linear in the range of 100–300 mg. The mean E_max_ of methylamine ranged from 19.167 to 124.970 ng/mL, with mean T_Emax_ ranging from 13.5 to 28.0 h. The complete inhibition (>90%) of SSAO activity was observed at 0.25–0.5 h post-dose and was maintained 48–168 h post-dose. In the multiple-ascending dose (MAD) study, a steady state was reached by day 5 in the 40 mg and 100 mg dose groups. Negligible accumulation was observed after repeated dosing. PK was linear in the range of 20–100 mg. Plasma methylamine appeared to plateau at doses of 20 mg and above, with mean E_max_ ranging from 124.142 to 156.070 ng/mL and mean T_Emax_ ranging from 14.2 to 22.0 h on day 7. SSAO activity in plasma was persistently inhibited throughout the treatment period. No evident change in methylamine and SSAO activity was observed in the placebo groups.

**Conclusion:** TT-01025-CL was safe and well-tolerated at a single dose of up to 300 mg and multiple doses of up to 100 mg once daily for 7 consecutive days. Absorption and elimination occurred rapidly in healthy volunteers. Linearity in plasma exposure was observed. TT-01025-CL inhibited SSAO activity rapidly and persistently in humans. The profile of TT-01025-CL demonstrates its suitability for further clinical development.

## 1 Introduction

Non-alcoholic fatty liver disease (NAFLD) is a condition defined by excessive fat accumulation in the form of triglycerides in the liver (Review Team et al., 2014), which has a global prevalence of 25% and is a leading cause of cirrhosis and hepatocellular carcinoma (Powell et al., 2021). Non-alcoholic steatohepatitis (NASH), a progressive form of NAFLD, is associated with liver damage, progressive fibrosis, cirrhosis, liver cancer, and death, affecting 3%–5% of the global population, with a higher prevalence among individuals with metabolic disorders such as insulin resistance and obesity (Rinella, 2015). Ahmed et al. (2012) reported a high incidence rate of NAFLD (>20%) among residents of Europe and the United States, which surpassed that of hepatitis C or alcoholic liver disease. Farrell et al. (2007) found that the incidence rate of NAFLD in the Asia–Pacific region remained between 12% and 24%. According to a global epidemiological survey, NAFLD’s incidence rate in China is approximately 15% (Forbes et al., 2011). NASH is an area with significant unmet medical needs.

The current treatment for NASH is limited to lifestyle changes and the management of co-morbidities. Regulatory agencies in most countries around the world, except India, have not yet approved drugs for the treatment of NASH (Fraile et al., 2021). However, a number of new treatments, which primarily target core pathways associated with NASH, including hepatocellular lipotoxicity, inflammation, and fibrosis, are being studied in late-stage clinical trials, and some of these have shown great promise (Sumida and Yoneda, 2018).

Vascular adhesion protein-1 (VAP-1), also known as semicarbazide-sensitive amine oxidase (SSAO), is a type 1 membrane-bound protein with a catalytic amine oxidase site proximal to the membrane. It is a member of the copper-containing amine oxidase family (Pannecoeck et al., 2015), which is associated with liver inflammation and fibrosis. VAP-1 catalyzes the oxidative deamination of primary amines, which results in the production of the corresponding aldehydes, hydrogen peroxide, and ammonium (Arndtz et al., 2017). VAP-1 is constitutively expressed in the human hepatic endothelium. It facilitates the recruitment of immune cells to the liver and increases oxidative stress through its metabolites (aldehydes and hydrogen peroxide) during liver inflammation, including NASH (Smith and Vainio, 2007). This function depends on the amine oxidase activity of VAP-1/SSAO. During the development of human NASH, both the circulating soluble form (sVAP-1) and the receptor form of VAP-1 are upregulated (Weston et al., 2015). The circulating sVAP-1 level is associated with disease severity and could be used to predict the presence of significant fibrosis in NASH, suggesting that sVAP-1 alone or in combination with other potential fibrosis markers could be used as biomarkers for NASH disease progression (Kurkijärvi et al., 1998; Weston and Adams, 2011). Given the role of VAP-1 in inflammatory diseases, inhibition of its activity offers a promising target for drug development (Danielli et al., 2022). In various preclinical NASH models, gene knockout or pharmacological inhibition of VAP-1 can reduce oxidative stress and recruitment of inflammatory cells to the liver and attenuate liver fibrosis (Foot et al., 2013; Weston et al., 2015). Current studies strongly support VAP-1 inhibition as an attractive independent therapeutic strategy for NASH.

NASH is advancing toward combination therapy (Powell et al., 2021). As liver inflammation is a key factor in the progression of NASH and related liver fibrosis (Xu et al., 2022), VAP-1 inhibitors can be used as promising anti-inflammatory agents (Lalor et al., 2007) that could be targeted in combination with other synergistic mechanisms, such as anti-fibrosis and lipid metabolism modulators. To date, no VAP-1 inhibitors have been commercially available. Investigations of several VAP-1 inhibitors are currently under development, with most of them in the clinical phase (Vakal et al., 2020).

TT-01025-CL [TransThera Sciences (Nanjing), Inc.] is a potent, highly selective, and irreversible VAP-1 inhibitor with low brain penetration. Preclinical studies have demonstrated its beneficial effects on steatosis, lobular inflammation, and liver fibrosis in NASH animal models. Here, we report a phase I clinical study for TT-01025-CL to assess its safety, tolerability, pharmacokinetic (PK), and pharmacodynamic (PD) characteristics in healthy Chinese adult subjects.

## 2 Methods

This was a phase 1 randomized, double-blind, placebo-controlled dose-escalation study designed to evaluate the safety, tolerability, pharmacokinetics, and pharmacodynamics of TT-01025-CL in healthy Chinese adult volunteers. The study protocol, amendments, and informed consent documentation were approved by the Ethics Committee of Nanjing Drum Tower Hospital, Affiliated Hospital of Medical School, Nanjing University. The study was conducted in accordance with Good Clinical Practice (GCP). This study (chinadrugtrials.org.cn CTR20211481) was conducted in accordance with international ethical guidelines, including the Declaration of Helsinki, International Council for Harmonisation of Technical Requirements for Pharmaceuticals for Human Use (ICH) guidelines, and other applicable laws and regulations. Written informed consent was obtained from all subjects.

### 2.1 Study design

In this escalation study, TT-01025-CL was administered orally in a fasted state. Safety and tolerability data were reviewed by the Safety Review Committee (SRC). Escalation to the next dose was allowed based on a review of emerging safety and PK data from the previous dose cohort. The study consisted of two parts.

Part 1: The single-ascending dose (SAD) study enrolled 36 healthy volunteers in five consecutive cohorts who received single oral doses of 10, 40, 100, 200, or 300 mg of TT-01025-CL. In the first cohort, four sentinel subjects received an oral dose of 10 mg TT-01025-CL. In the remaining four cohorts, eight subjects in each cohort were randomly assigned in a 3:1 allocation ratio to receive a single dose of 40, 100, 200, or 300 mg TT-01025-CL or a matching placebo in a fasted state to assess safety and tolerability. PK and PD samples were collected throughout the study. The SAD study process is briefly described in Supplementary Figure S1. The dose escalation meeting should be held after obtaining at least D8 and previous visit safety and tolerability data.

Part 2: The multiple-ascending dose (MAD) study enrolled 24 subjects in three separate cohorts. Eight healthy subjects in each cohort were randomly assigned in a 3:1 ratio to receive 20, 40, or 100 mg of TT-01025-Cl or a matching placebo orally once daily for seven consecutive days in a fasted state. Safety and tolerability were assessed. PK and PD samples were collected after administration. The doses for the MAD study were chosen based on safety and PK/PD data from the SAD study. The MAD study process is briefly described in Supplementary Figure S2. The dose escalation meeting should be held after obtaining at least D14 and previous visit safety and tolerability data.

### 2.2 Subject selection

Subjects were healthy volunteers aged 18–45 years [no pregnant or breastfeeding; contraception for at least 90 days before screening; body mass index (BMI), 18–28 kg/m^2^; male body weight ≥50.0 kg; and female body weight ≥45.0 kg]. Strict contraception should be used during the study period and for 90 days after the last administration. Smoking should be less than 10 cigarettes the day before screening, and the average daily alcohol intake should be less than 15 g in the week prior to screening. No prescription, over-the-counter, or Chinese herbal medicines were allowed within 14 days prior to study drug administration.

Subjects were excluded if they had impaired cardiac function, including clinically significant arrhythmias or other clinically detected abnormalities, including, but not limited to 1) pulse <50 beats per minute (bpm) or >100 bpm; 2) systolic blood pressure (SBP) < 90 mmHg or >140 mmHg or diastolic blood pressure (DBP) was <50 mmHg or >90 mmHg; 3) the QT interval (QTcF) corrected using the Fridericia’s formula was >450 ms; and 4) ECG showing atrioventricular block of second degree or higher. Subjects were excluded if they had impaired kidney function or abnormal liver enzymes, including, but not limited to 1) alanine aminotransferase (ALT) > 2× upper limit of normal value (ULN); 2) aspartate aminotransferase (AST) > 2×ULN; and 3) eGFR was <90 mL/min/1.73 m^2^.

### 2.3 Safety assessments

The primary objective of this study was to assess the safety and tolerability of TT001025-CL, that is, the incidence and severity of adverse events (AEs) (rated as mild, moderate, and severe), including subjects’ main complaint, physical examination, vital signs, 12-lead electrocardiogram, ultrasound examination of testicles and epididymis, and clinical laboratory evaluation. The AEs were coded using the Medical Dictionary for Regulatory Activities (MedDRA), version 24.0.

### 2.4 Pharmacokinetic analyses

In the SAD study, blood samples were collected at pre-dose (0 h) and 0.25, 0.5, 1, 2, 4, 6, 8, 12, 24, and 48 h after dose administration. In the MAD study, serial blood samples were collected at pre-dose (0 h) on study days 1, 3, 4, 5, 6, and 7; at 0.25, 0.5, 1, 2, 4, 6, 8, 12, and 24 h after administration on days 1 and 7; and at 48 h after administration on day 7. Plasma concentrations of TT-01025-CL were measured using a validated liquid chromatography-tandem mass spectrometry (LC-MS/MS) method. A stable isotope-labeled (deuterated) internal standard, TT-01411-CL, was used. The lower limit of quantitation for TT-01025-CL was 1.00 ng/mL, with a calibration range of 1.00–1000 ng/mL. The precision [coefficient of variation (CV)] values in validation were within 6.0%; the accuracy values were −3.2%–2.1%. Pharmacokinetic parameters were calculated using non-compartmental analysis (NCA) using a validated Phoenix WinNonlin 8.3.

The following PK parameters were calculated: area under the plasma drug concentration–time curve (AUC_0-t_ and AUC_0-∞_), maximum plasma concentration (C_max_), time to reach maximum plasma concentration (T_max_), terminal elimination half-life (t_1/2z_), apparent clearance (CL_z_/F), apparent volume of distribution (V_z_/F), and mean retention time (MRT_0-t_ and MRT_0-∞_). The steady-state pharmacokinetic parameters of TT-01025-Cl were derived, including area under the concentration–time curve at steady state (AUC_0-t,ss_, AUC_τ,ss_, and AUC_0-∞,ss_), maximum plasma concentration at steady state (C_max,ss_), plasma minimum concentration at steady state (C_min,ss_), plasma trough concentration (C_trough_), time to reach maximum plasma concentration at steady state (T_max,ss_), apparent clearance rate at steady state (CL_ss_/F), apparent volume of distribution (V_z_/F), terminal half-life (T_1/2z_), accumulation ratio based on AUC [R_ac (AUC)_], accumulation ratio based on C_max_ (R_ac (Cmax)_), and degree of fluctuation (DF).

### 2.5 Pharmacodynamic analyses

In the SAD study, PD biomarker measurements were performed on blood samples collected at pre-dose and 0.25, 0.5, 1, 2, 4, 6, 8, 12, 24, 48, and 168 h after dose administration. In the MAD study, serial plasma PD samples were collected at pre-dose on study days 1, 3, 4, 5, 6, and 7; at 0.25, 0.5, 1, 2, 4, 6, 8, 12, and 24 h after administration on days 1 and 7; and at 48 h and 168 h after administration on day 7.

Plasma SSAO-specific amine oxidase activity and plasma methylamine accumulation were assessed as PD biomarkers of target engagement. In the plasma SSAO-specific activity assay, pargyline (10 µL, 10 mmol/L in water), a potent and selective MAO-A and B inhibitor, was added to the sample to a final concentration of 0.5 mmol/L. At this concentration, pargyline can completely inhibit the endogenous MAO-A/B enzyme without affecting SSAO activity. SSAO-specific activity in plasma was determined using a fluorometric method to detect hydrogen peroxide (H_2_O_2_) generation upon the addition of benzylamine as a substrate. Percent changes were calculated relative to baseline samples additionally treated with a high dose (60 nmol/L) of TT-01025-CL, which served as a background control. The average inhibition rates of HQC/MQC/LQC subsequent acceptance standards were 73.77%–123.77%, 56.28%–106.28%, and −25.00%–25.00%, respectively. Methylamine was an endogenous substrate of SSAO, and plasma methylamine was expected to increase upon SSAO inhibition. Plasma methylamine accumulation was determined using the LC-MS/MS method. Plasma samples were thawed on ice, and a working internal standard (20 µL of 250 ng/mL of methyl-13C, D3-amine) was added and vortexed, followed by the addition of 300 µL of acetonitrile (ACN) and centrifugation. Then, 100 μL of supernatants were transferred to a 96-well plate, followed by the successive addition of 50 µL of 0.1 mol/L sodium tetraborate in water and 100 µL of 2% benzol chloride in ACN. Samples were stored at room temperature for 10 min before adding 100 µL of 20% formic acid in water and 300 µL of ACN centrifuged at 3500 rpm for 3 min Then, 550 μL of supernatants were transferred to a new 96-well plate, and 150 µL of 0.25 mol/L ammonium formate solution was added and vortexed at 1000 rpm for 1 min Afterward, 5 μL of each sample was subjected to LC-MS/MS analysis. The lower limit of quantitation was 5.00 ng/mL, and the calibration curve ranged from 5.00 to 500 ng/mL. The precision (CV) of the assay was within 2.3%, and the accuracy was between −6.8% and −0.5%.

### 2.6 Statistical analyses

Descriptive statistics were calculated for safety, PK, and PD measures. Safety data were analyzed in the safety analysis set, which included all subjects who received at least one dose of the study drug. PK data were analyzed for the pharmacokinetic analysis set using Phoenix WinNonlin 8.3.3, which included all subjects who received at least one dose of the study drug and had at least one active PK parameter during the study. Statistical testing was two-sided and used the 95% significance level. Non-compartmental methods were used to estimate PK parameters for all analytes with sufficient data above the LLOQ from the concentration–time profiles for the PK analysis set. A regression power model, relating log-transformed C_max_ and AUC parameters to log-transformed dose, was used to investigate dose proportionality. Individual and geometric mean dose-normalized C_max_, AUC_0-t_, and AUC_0-∞_ were plotted against dose levels. PD data were analyzed for the pharmacodynamic analysis set, which included all subjects who received one or more doses of the study drug and had at least one post-dose PD evaluation dataset, and summarized using descriptive statistics.

## 3 Results

### 3.1 Subject characteristics

A total of 60 healthy subjects were enrolled in the study, with 36 subjects in the SAD cohorts and 24 in the MAD cohorts. The demographic and baseline characteristics were generally well-matched across treatment groups. The demographic characteristics of the subjects are summarized in Tables 1, 2.

**TABLE 1:** Demographics of the single-ascending dose study.

Characteristic	Placebo	10 mg	40 mg	100 mg	200 mg	300 mg	Total (TT-01025-CL)	Total
N	8	4	6	6	6	6	28	36
Age (yr)	25.6 ± 5.68	32.3 ± 9.50	27.2 ± 5.04	25.2 ± 8.93	29.8 ± 3.97	31.0 ± 4.90	28.9 ± 6.59	28.1 ± 6.47
Height (cm)	167.25 ± 7.076	161.88 ± 10.858	161.75 ± 8.299	172.08 ± 9.457	167.08 ± 6.771	166.92 ± 5.352	166.23 ± 8.448	166.46 ± 8.078
Weight (kg)	61.80 ± 7.283	60.85 ± 8.682	59.08 ± 8.979	66.97 ± 14.844	66.95 ± 4.818	64.20 ± 3.958	63.81 ± 9.056	63.36 ± 8.637
BMI (kg/m^2^)	22.09 ± 2.392	23.13 ± 0.574	22.48 ± 1.891	22.53 ± 4.054	24.03 ± 2.110	23.12 ± 2.112	23.05 ± 2.396	22.84 ± 2.396
Male, n (%)	5 (62.5)	1 (25.0)	1 (16.7)	3 (50.0)	4 (66.7)	4 (66.7)	13 (46.4)	18 (50.0)
Female, n (%)	3 (37.5)	3 (75.0)	5 (83.3)	3 (50.0)	2 (33.3)	2 (33.3)	15 (53.6)	18 (50.0)
Alcohol history								
Non-drinker, n (%)	7 (87.5)	4 (100.0)	6 (100.0)	6 (100.0)	4 (66.7)	5 (83.3)	25 (89.3)	32 (88.9)
Drinker, n (%)	1 (12.5)	0 (0)	0 (0)	0 (0)	2 (33.3)	1 (16.7)	3 (10.7)	4 (11.1)
Smoking history								
Non-smoker, n (%)	5 (62.5)	4 (100.0)	6 (100.0)	6 (100.0)	5 (83.3)	6 (100.0)	27 (96.4)	32 (88.9)
Smoker, n (%)	3 (37.5)	0 (0)	0 (0)	0 (0)	1 (16.7)	0 (0)	1 (3.6)	4 (11.1)

Age, height, weight, and BMI are presented as the mean (standard deviation).

Abbreviations: BMI, body mass index.

**TABLE 2:** Demographics of the multiple-ascending dose study.

Characteristic	Placebo	20 mg	40 mg	100 mg	Total (TT-01025-CL)	Total
N	6	6	6	6	18	24
Age (yr)	26.5 ± 3.78	26.0 ± 4.98	31.7 ± 6.31	28.3 ± 6.47	28.7 ± 6.09	28.1 ± 5.61
Height (cm)	165.50 ± 10.060	169.25 ± 6.089	162.75 ± 8.948	168.25 ± 7.182	166.75 ± 7.634	166.44 ± 8.086
Weight (kg)	61.88 ± 10.024	67.78 ± 10.037	63.53 ± 9.498	66.02 ± 8.499	65.78 ± 8.979	64.80 ± 9.187
BMI (kg/m^2^)	22.48 ± 1.747	23.57 ± 2.593	23.88 ± 1.831	23.32 ± 2.782	23.59 ± 2.302	23.31 ± 2.195
Male, n (%)	5 (83.3)	5 (83.3)	2 (33.3)	4 (66.7)	11 (61.1)	16 (66.7)
Female, n (%)	1 (16.7)	1 (16.7)	4 (66.7)	2 (33.3)	7 (38.9)	8 (33.3)
Alcohol history						
Non-drinker, n (%)	5 (83.3)	5 (83.3)	6 (100.0)	6 (100.0)	17 (94.4)	22 (91.7)
Drinker, n (%)	1 (16.7)	1 (16.7)	0 (0)	0 (0)	1 (5.6)	2 (8.3)
Smoking history						
Non-smoker, n (%)	5 (83.3)	6 (100.0)	5 (83.3)	6 (100.0)	17 (94.4)	22 (91.7)
Smoker, n (%)	1 (16.7)	0 (0)	1 (16.7)	0 (0)	1 (5.6)	2 (8.3)

Age, Height, weight, and BMI are presented as the mean (standard deviation).

Abbreviations: BMI, body mass index.

### 3.2 Safety and tolerability

In this study, TT-01025-CL was well-tolerated in all dosing cohorts of healthy Chinese subjects. No serious AEs were reported, and no subject discontinued due to an AE. All treatment-emergent adverse events (TEAEs) were mild and moderate in intensity. No dose-dependent increase in the intensity or frequency of AEs was observed.

In the SAD study, at least one TEAE was reported in 9 of 28 (32.1%) healthy subjects who received TT-01025-CL and 4 of 8 (50.0%) subjects who received a placebo. The most frequently reported TEAEs were infections and infectious diseases (upper respiratory tract infection and blepharitis), which occurred in both the treatment and placebo groups (14.3% and 25.0%, respectively). The incidence of all TEAEs by system organ class (SOC) and preferred term (PT) for the SAD study is shown in Table 3. The AEs occurring in at least 10% of participants in the TT-01025-CL group were upper respiratory tract infection (10.7%) and increased blood uric acid (14.3%).

**TABLE 3:** Summary of TEAEs after single-dose administration.

**SOC** PT	Placebo		10 mg		40 mg		100 mg		200 mg		300 mg		Total (TT-01025-CL)		Total	
	Case	Time	Case	Time	Case	Time	Case	Time	Case	Time	Case	Time	Case	Time	Case	Time
N	8		4		6		6		6		6		28		36	
Total n (%)	4 (50.0)	5	3 (75.0)	6	1 (16.7)	1	1 (16.7)	1	1 (16.7)	1	3 (50.0)	3	9 (32.1)	12	13 (36.1)	17
**Infections and infestations**, n (%)	2 (25.0)	2	2 (50.0)	3	0 (0)	0	0 (0)	0	0 (0)	0	2 (33.3)	2	4 (14.3)	5	6 (16.7)	7
Upper respiratory tract infection, n (%)	2 (25.0)	2	1 (25.0)	1	0 (0)	0	0 (0)	0	0 (0)	0	2 (33.3)	2	3 (10.7)	3	5 (13.9)	5
Blepharitis, n (%)	0 (0)	0	1 (25.0)	2	0 (0)	0	0 (0)	0	0 (0)	0	0 (0)	0	1 (3.6)	2	1 (2.8)	2
**Investigations n** (%)	2 (25.0)	2	0 (0)	0	1 (16.7)	1	1 (16.7)	1	1 (16.7)	1	1 (16.7)	1	4 (14.3)	4	6 (16.7)	6
Increased blood uric acid, n (%)	2 (25.0)	2	0 (0)	0	1 (16.7)	1	1 (16.7)	1	0 (0)	0	0 (0)	0	2 (7.1)	2	4 (11.1)	4
Increased blood cholesterol, n (%)	0 (0)	0	0 (0)	0	0 (0)	0	0 (0)	0	0 (0)	0	1 (16.7)	1	1 (3.6)	1	1 (2.8)	1
Increased blood triglycerides, n (%)	0 (0)	0	0 (0)	0	0 (0)	0	0 (0)	0	1 (16.7)	1	0 (0)	0	1 (3.6)	1	1 (2.8)	1
**Injury, poisoning, and procedural complications,** n (%)	0 (0)	0	1 (25.0)	1	0 (0)	0	0 (0)	0	0 (0)	0	0 (0)	0	1 (3.6)	1	1 (2.8)	1
Animal bite, n (%)	0 (0)	0	1 (25.0)	1	0 (0)	0	0 (0)	0	0 (0)	0	0 (0)	0	1 (3.6)	1	1 (2.8)	1
**Skin and subcutaneous tissue disorders,** n (%)	0 (0)	0	1 (25.0)	1	0 (0)	0	0 (0)	0	0 (0)	0	0 (0)	0	1 (3.6)	1	1 (2.8)	1
Allergic dermatitis, n (%)	0 (0)	0	1 (25.0)	1	0 (0)	0	0 (0)	0	0 (0)	0	0 (0)	0	1 (3.6)	1	1 (2.8)	1
**Gastrointestinal disorders,** n (%)	0 (0)	0	1 (25.0)	1	0 (0)	0	0 (0)	0	0 (0)	0	0 (0)	0	1 (3.6)	1	1 (2.8)	1
Diarrhea, n (%)	0 (0)	0	1 (25.0)	1	0 (0)	0	0 (0)	0	0 (0)	0	0 (0)	0	1 (3.6)	1	1 (2.8)	1
**Cardiac disorders,** n (%)	1 (12.5)	1	0 (0)	0	0 (0)	0	0 (0)	0	0 (0)	0	0 (0)	0	0 (0)	0	1 (2.8)	1
Sinus bradycardia, n (%)	1 (12.5)	1	0 (0)	0	0 (0)	0	0 (0)	0	0 (0)	0	0 (0)	0	0 (0)	0	1 (2.8)	1

Abbreviations: TEAE, treatment-emergent adverse event; SOC, system organ class; PT, preferred term.

In the MAD study, at least one TEAE was reported in 10 of 18 (55.6%) in the TT-01025-CL groups and 1 of 6 (16.7%) in the placebo groups. The most common TEAEs in subjects were for various investigations, occurring in both the TT-01025-CL and placebo groups at an incidence of 33.3% and 16.7%, respectively. The details are shown in Table 4. The AEs occurring in at least 10% of participants in the TT-01025-CL group were abnormal ECG T-waves (16.7%), increased blood triglyceride (16.1%), diarrhea (11.1%), and sinus bradycardia (11.1%).

**TABLE 4:** Summary of TEAEs after multiple-dose administration.

**SOC** PT	Placebo		20 mg		40 mg		100 mg		Total (TT-01025-CL)		Total	
	Case	Time	Case	Time	Case	Time	Case	Time	Case	Time	Case	Time
N	6		6		6		6		18		24	
Total n (%)	1 (16.7)	1	4 (66.7)	7	4 (66.7)	6	2 (33.3)	2	10 (55.6)	15	11 (45.8)	16
**Investigations,** n (%)	1 (16.7)	1	3 (50.0)	4	2 (33.3)	2	1 (16.7)	1	6 (33.3)	7	7 (29.2)	8
Electrocardiogram T wave abnormal, n (%)	0 (0)	0	1 (16.7)	1	1 (16.7)	1	1 (16.7)	1	3 (16.7)	3	3 (12.5)	3
Increased blood triglyceride, n (%)	1 (16.7)	1	2 (33.3)	2	0 (0)	0	0 (0)	0	2 (11.1)	2	3 (12.5)	3
Decreased white blood cell count, n (%)	0 (0)	0	0 (0)	0	1 (16.7)	1	0 (0)	0	1 (5.6)	1	1 (4.2)	1
Increased blood uric acid, n (%)	0 (0)	0	1 (16.7)	1	0 (0)	0	0 (0)	0	1 (5.6)	1	1 (4.2)	1
**Gastrointestinal ** **disorders,** n (%)	0 (0)	0	1 (16.7)	1	1 (16.7)	2	0 (0)	0	2 (11.1)	3	2 (8.3)	3
Diarrhea, n (%)	0 (0)	0	1 (16.7)	1	1 (16.7)	1	0 (0)	0	2 (11.1)	2	2 (8.3)	2
Abdominal pain, n (%)	0 (0)	0	0 (0)	0	1 (16.7)	1	0 (0)	0	1 (5.6)	1	1 (4.2)	1
**Cardiac disorders,** n (%)	0 (0)	0	1 (16.7)	2	0 (0)	0	1 (16.7)	1	2 (11.1)	3	2 (8.3)	3
Sinus bradycardia, n (%)	0 (0)	0	1 (16.7)	2	0 (0)	0	1 (16.7)	1	2 (11.1)	3	2 (8.3)	3
**Skin and subcutaneous ** **tissue disorders,** n (%)	0 (0)	0	0 (0)	0	1 (16.7)	2	0 (0)	0	1 (5.6)	2	1 (4.2)	2
Eczema, n (%)	0 (0)	0	0 (0)	0	1 (16.7)	2	0 (0)	0	1 (5.6)	2	1 (4.2)	2

Abbreviations: TEAE, treatment-emergent adverse event; SOC, system organ class; PT, preferred term.

In all volunteers, there were no clinically significant findings that were reported in physical and ultrasound examinations at any preset time points. Compared with baseline, there was no significant increase in the frequency of abnormal results in vital signs and ECG. No significant changes in the mean laboratory test values were detected from the results of laboratory tests, including routine blood, blood biochemistry, urine, and coagulation.

### 3.3 Pharmacokinetic properties

All 60 volunteers who received TT-01025-CL administration were included in the PK analysis.

#### 3.3.1 SAD

**FIGURE 1:**
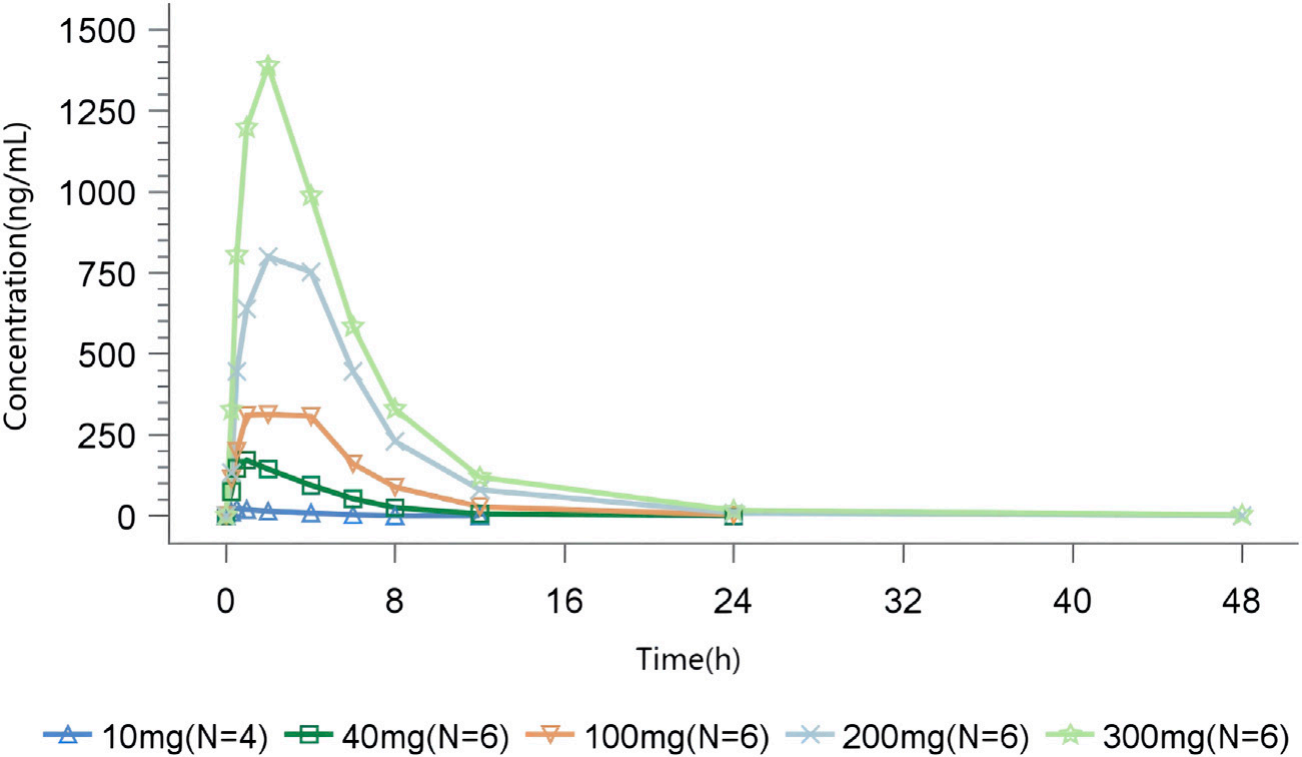
Mean TT-01025-CL plasma concentration–time profile after single-dose administration.

In the SAD study, after a single oral administration, TT-01025-CL was rapidly absorbed, with a median time to reach T_max_ ranging from 0.5 to 2 h across different dose groups. In the 10 mg to 200 mg dose group, the time to peak was progressively delayed as the dose was increased. The mean C_max_, mean AUC_0-t_, and AUC_0-∞_ values in plasma increased in a slightly greater-than-dose-proportional manner within the range of 10–300 mg (Figure 1, Table 5). Dose proportionality was concluded since the lower limit of the 95% CI for the slope of ln (C_max_) (β = 1.195 [1.053–1.338]), ln (AUC_0-t_) (β = 1.384 [1.236–1.531]), and ln (AUC_0-∞_) (β = 1.359 [1.221–1.497]) to ln (dose) was greater than 1 within the range of 10 mg–200 mg, 10 mg–100 mg, and 40 mg–300 mg, respectively. PK was linear in the range of 100 mg–300 mg, as the 95% CIs for the slopes of ln (C_max_) (β = 1.192 [0.837–1.547]), ln (AUC_0-t_) (β = 1.265 [0.938–1.591]), and ln (AUC_0-∞_) (β = 1.260 [0.938–1.582]) on ln (dose) included the value of 1 (Supplementary Table S1; Supplementary Figure S3). The mean clearance (CL_z_/F) and volume of distribution (V_z_/F) decreased with the increase in dose, indicating that the elimination time of TT-01025-CL was prolonged with the increase in doses.

**TABLE 5:** Summary of pharmacokinetic parameters of TT-01025-CL after single-dose administration.

Parameter	10 mg (*n* = 4)	40 mg (*n* = 6)	100 mg (*n* = 6)	200 mg (*n* = 6)	300 mg (*n* = 6)
C_max_ (ng/mL)	23.33 ± 15.33	178.56 ± 55.22	387.02 ± 130.32	874.39 ± 285.52	1,418.79 ± 370.09
AUC_0-t_ (h*ng/mL)	81.25 ± 66.04	790.60 ± 246.44	2,144.22 ± 677.46	5,412.97 ± 1524.97	8,265.57 ± 1,748.90
AUC_0-∞_(h*ng/mL)	85.86 ± 64.34	805.06 ± 247.10	2,165.86 ± 676.15	5,440.92 ± 1,519.94	8,315.73 ± 1,732.27
T_max_(h)	0.50 (0.50, 0.50)	1.00 (0.5, 2)	1.50 (0.5, 4)	2.00 (1, 4.02)	2.00 (1, 4)
t_1/2z_(h)	2.23 ± 0.68	2.09 ± 0.44	3.07 ± 0.90	3.71 ± 1.49	4.39 ± 0.88
V_z_/F(L)	599.80 ± 480.66	159.16 ± 48.26	217.28 ± 67.96	209.31 ± 88.67	230.13 ± 24.04
CL_z_/F(L/h)	167.90 ± 100.48	54.58 ± 19.90	51.09 ± 19.63	40.00 ± 14.52	37.51 ± 8.44
λ_z_(1/h)	0.34 ± 0.12	0.34 ± 0.06	0.24 ± 0.06	0.20 ± 0.05	0.16 ± 0.03
MRT_0-t_(h)	2.94 ± 0.41	3.57 ± 0.69	4.72 ± 0.87	5.30 ± 0.56	5.22 ± 0.85
MRT_0-∞_(h)	3.77 ± 1.08	3.79 ± 0.71	4.94 ± 0.99	5.45 ± 0.62	5.40 ± 0.86

Data are presented as the mean (standard deviation) except for T_max_, which is shown as the median (min, max).

Abbreviations: C_max_, maximum concentration; AUC_0-t_, area under the concentration–time curve from time zero to the last quantifiable plasma concentration; AUC_0-∞_, area under the concentration–time curve from time zero extrapolated to infinity; T_max_, time to reach maximum concentration; t_1/2z_, terminal half-life; Vz/F, apparent volume of distribution; CL_z_/F, apparent clearance; λz, elimination rate constant; MRT_0-t_, mean retention time from time zero to the last quantifiable plasma concentration; MRT_0-∞_, mean retention time from time zero extrapolated to infinity.

#### 3.3.2 MAD

**FIGURE 2:**
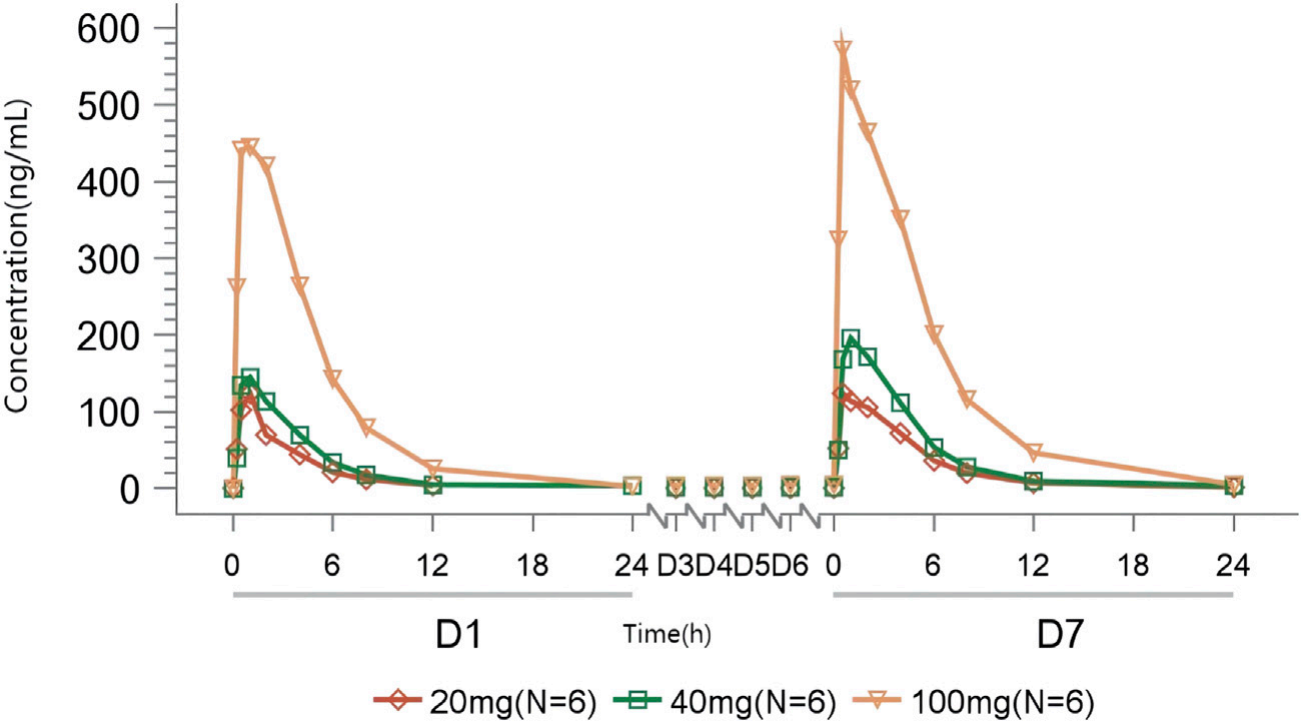
Mean TT-01025-CL plasma concentration–time profile after multiple-dose administration.

Rapid absorption and elimination of TT-01025-CL were also observed in the MAD study (Figure 2). Steady-state conditions were reached by day 5 (Supplementary Table S2). Steady-state analysis could not be performed in the 20 mg group since only three measurable blood concentrations were available before day 5–7 administration. Median T_max_ ranged from 0.75 to 1 h and 0.5–1 h on days 1 and 7, respectively. Mean t_1/2z_ ranged from 2.17 to 3.24 h and 2.54–3.37 h on days 1 and 7, respectively. C_max_ and AUC increased in a dose-dependent manner, while no dose-dependent trend was observed in T_max_, t_1/2z_, V_z_/F, and CL_z_/F on days 1 and 7. Rac_(Cmax)_ was 1.10, 1.49, and 1.24, and Rac_(AUC)_ was 1.54, 1.56, 1.32, respectively (Table 6), implying no plasma accumulation of TT-01025-CL on day 7. Based on estimates of the exponent from the power model, 95% CI for the slope ln(C_max,ss_) (β = 0.927 [0.603–1.251]), ln(AUC_0-t,ss_) (β = 1.133 [0.878–1.387]), and ln(AUC_0-∞,ss_) (β = 1.119 [0.862–1.375]) on ln(dose) included the value of 1, indicating that PK was linear within the range of 20 mg–100 mg (Supplementary Table S3; Supplementary Figure S4).

**TABLE 6:** Summary of pharmacokinetic parameters of TT-01025-CL after multiple-dose administration.

Parameter	20 mg (*n* = 6)	40 mg (*n* = 6)	100 mg (*n* = 6)
D1			
C_max_ (ng/mL)	132.92 ± 51.66	165.91 ± 55.49	574.44 ± 219.79
AUC_0-t_ (h*ng/mL)	405.81 ± 133.74	600.41 ± 190.28	2,353.64 ± 444.12
AUC_0-∞_(h*ng/mL)	416.88 ± 136.38	614.10 ± 203.96	2,366.33 ± 449.12
T_max_(h)	1.00 (0.5,1)	1.00 (0.5,2)	0.75 (0.23,4)
t_1/2z_(h)	2.17 ± 0.16	2.18 ± 1.02	3.24 ± 0.33
V_z_/F(L)	166.74 ± 61.75	205.68 ± 56.26	203.38 ± 45.29
CL_z_/F (L/h)	52.52 ± 16.75	71.14 ± 22.23	43.64 ± 8.80
λ_z_ (1/h)	0.32 ± 0.02	0.36 ± 0.11	0.22 ± 0.02
MRT_0-t_(h)	3.12 ± 0.23	3.41 ± 0.96	4.21 ± 0.79
MRT_0-∞_, (h)	3.44 ± 0.27	3.68 ± 1.20	4.34 ± 0.83
D7			
C_max,ss_ (ng/mL)	132.30 ± 22.30	224.11 ± 20.59	627.20 ± 104.44
C_min,ss_ (ng/mL)	0.19 ± 0.48	0.80 ± 1.29	4.27 ± 2.88
C_trough_ (ng/mL)	0.78 ± 0.67	1.12 ± 1.66	4.86 ± 2.23
AUC_0-t,ss_ (h*ng/mL)	604.02 ± 122.24	923.74 ± 255.60	3,071.47 ± 452.58
AUC_0-∞,ss_ (h*ng/mL)	619.68 ± 120.17	940.67 ± 260.80	3,095.68 ± 462.82
AUC_τ,ss_ (AUC_0–24h_) (h*ng/mL)	614.93 ± 118.55	933.26 ± 250.17	3,071.26 ± 452.53
T_max,ss_(h)	0.5 (0.5, 2)	1.00 (0.5, 2)	0.5 (0.5, 1)
t_1/2z_(h)	3.23 ± 1.01	2.54 ± 1.06	3.37 ± 0.35
DF (%)	523.40 ± 88.73	600.69 ± 121.55	489.05 ± 63.17
V_z_/F(L)	154.26 ± 50.53	153.63 ± 27.88	160.48 ± 24.92
CL_ss_/F (L/h)	33.61 ± 6.80	45.42 ± 11.62	33.14 ± 4.78
λ_z_ (1/h)	0.23 ± 0.08	0.31 ± 0.11	0.21 ± 0.02
MRT_0-∞, ss_(h)	4.46 ± 0.58	4.08 ± 1.22	4.82 ± 0.45
Ra_(Cmax)_	1.10 ± 0.40	1.49 ± 0.52	1.24 ± 0.49
Ra_(AUC)_	1.54 ± 0.25	1.56 ± 0.16	1.32 ± 0.09

Data are presented as the mean (standard deviation), except for T_max_, which is shown as the median (min, max).

Abbreviations: C_max_, maximum concentration; AUC_0-t_, area under the concentration–time curve from time zero to the last quantifiable concentration; AUC_0-∞_, area under the concentration–time curve from time zero extrapolated to infinity; T_max_, time to reach maximum concentration; t_1/2z_, terminal half-life; DF: degree of fluctuation; Vz/F, apparent volume of distribution; CL_z_/F, apparent clearance; λ_z_, elimination rate constant; MRT_0-t_, mean retention time from time zero to the last quantifiable plasma concentration; MRT_0-∞_, mean retention time from time zero extrapolated to infinity; C_max,ss_, maximum concentration at the steady state; C_min,ss_, minimum concentration at the steady state; C_trough_, trough concentration. AUC_0-t,ss:_ area under the concentration–time curve from time zero to the last quantifiable concentration at the steady state.; AUC_0-∞, ss_, area under the concentration–time curve from time zero extrapolated to infinity at the steady state; AUC_τ, ss_ (AUC_0–24 h_), area under the concentration–time curve at one dosing interval (0–24 h) at the steady state; MRT_0-∞,ss_, mean retention time from time zero extrapolated to infinity at the steady state. Ra_(Cmax)_, accumulation ratio based on C_max_; Ra_(AUC)_, accumulation ratio based on AUC.

### 3.4 Pharmacodynamic properties

As an irreversible inhibitor, the key PD endpoint is the proportion of the target occupied by TT-01025-CL. Two PD biomarkers, plasma SSAO-specific amine oxidase activity and plasma methylamine accumulation, were investigated.

#### 3.4.1 SAD

**FIGURE 3:**
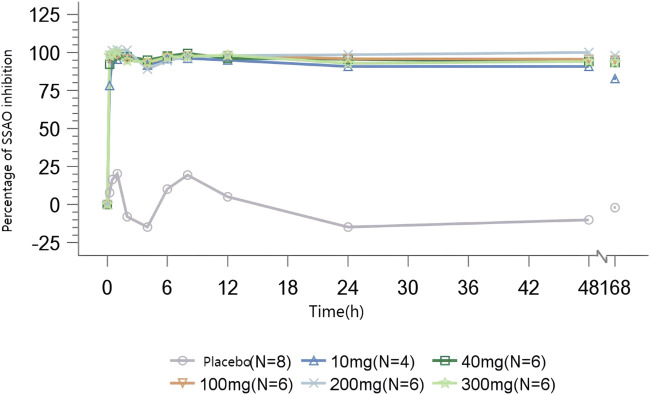
Mean percentage of SSAO activity inhibition after single-dose administration.

In volunteers administrated with TT-01025-CL, rapid and sustained inhibition of plasma SSAO-specific amine oxidase activity was observed following a single dose. In the range of 10–300 mg of TT-01025-CL, complete inhibition (>90%) was observed at 0.25–0.5 h post-dose and was maintained after the plasma concentration of TT-01025-CL fell below the LLOQ (48–168 h post-dose). There was no evident inhibition of SSAO-specific amine oxidase activity in the placebo groups (Figure 3).

**FIGURE 4:**
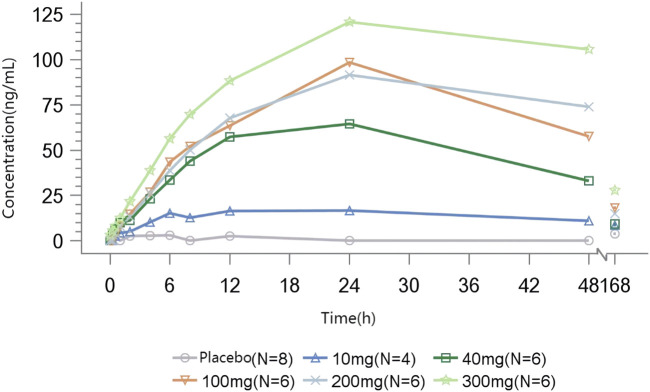
Mean methylamine concentration–time profile after single-dose administration.

The mean E_max_ of methylamine ranged from 19.167 to 124.970 ng/mL. A dose-dependent increase in plasma methylamine was observed following a single dose of TT-01025-CL, approaching maximum response at a dose of 100 mg and above. Plasma methylamine reached its peak at 12–24 h after administration, returning to baseline over 7 days post-dose. There was no evident change in methylamine in the placebo groups (Figure 4; Table 7).

**TABLE 7:** Summary of parameters of methylamine after single-dose administration.

	Placebo	10 mg (*n* = 4)	40 mg (*n* = 6)	100 mg (*n* = 6)	200 mg (*n* = 6)	300 mg (*n* = 6)
E_max_ (ng/mL)	2.179 ± 3.013	19.167 ± 2.800	66.047 ± 10.364	98.529 ± 28.020	91.484 ± 25.051	124.970 ± 29.466
T_Emax_(h)	0 (0∼168)	12.00 (6∼24)	24.00 (12∼24)	24.00 (24∼24)	24.00 (24∼24)	24.00 (24∼48)
AUEC_0–24h_ (h*ng/mL)	9.653 ± 22.883	330.043 ± 30.826	1,115.562 ± 245.351	1,425.227 ± 350.457	1,396.393 ± 396.360	1,875.492 ± 393.861
AUEC_0–48h_ (h*ng/mL)	9.653 ± 22.883	663.530 ± 60.116	2,286.867 ± 367.199	3,297.417 ± 854.453	3,382.727 ± 959.800	4,593.392 ± 1,131.910
AUEC_0–168h_ (h*ng/mL)	96.345 ± 179.447	1,835.675 ± 471.450	4,824.267 ± 1,031.561	7,836.257 ± 2,838.663	8,730.057 ± 3,120.966	12,604.802 ± 4,111.660

Data are presented as the mean (standard deviation), except for T_Emax_, which is shown as the median (min, max).

Abbreviations: E_max_, maximum concentration of methylamine; T_Emax_, time to reach maximum concentration of methylamine; AUEC_0–24 h (48 h, 168 h),_ area under the concentration–time curve from time zero to 24 h (48 h, 168 h).

#### 3.4.2 MAD

**FIGURE 5:**
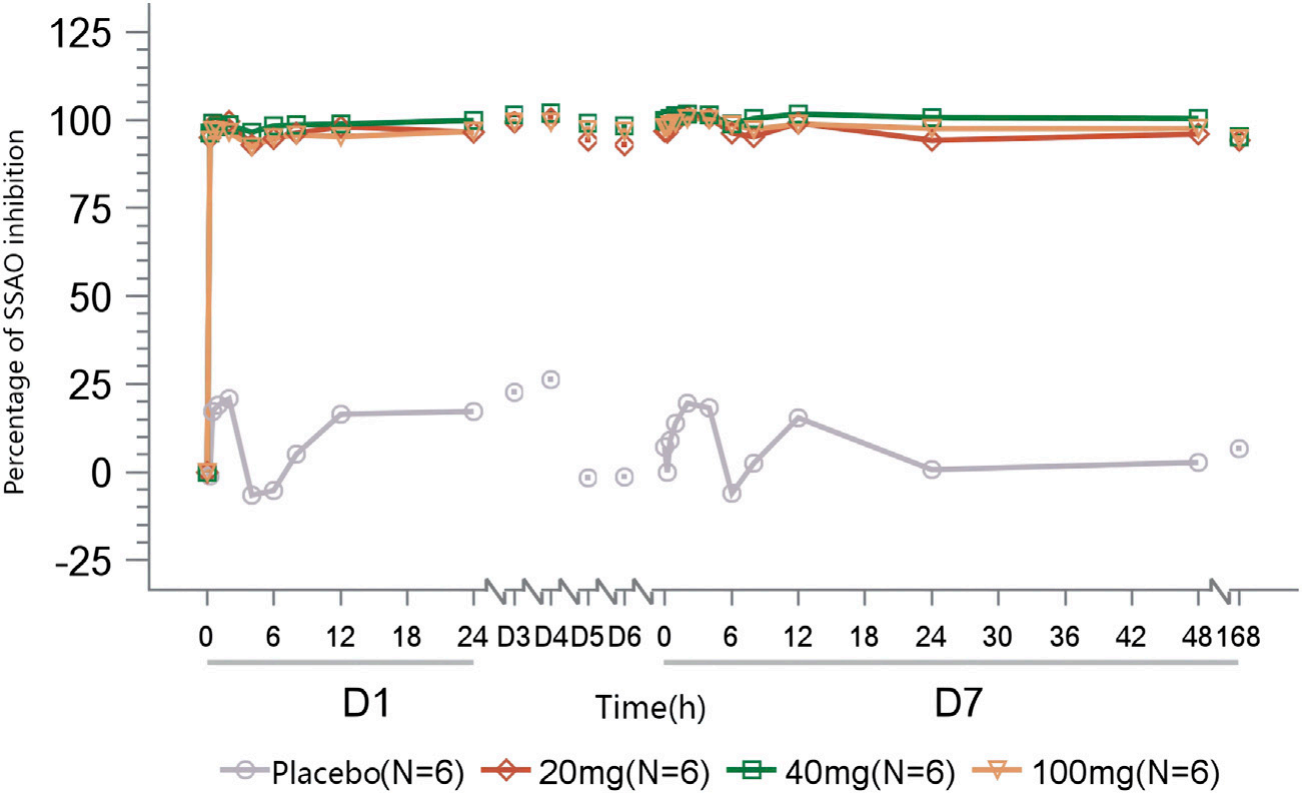
Mean percentage of SSAO activity inhibition after multiple-dose administration.

In subjects administered multiple doses of TT-01025-CL, rapid and sustained inhibition (>90%) of plasma SSAO-specific amine oxidase activity was observed on day 1 of administration and sustained until 168 h after the last administration on day 7. No notable inhibition was observed in the placebo subjects (Figure 5).

**FIGURE 6:**
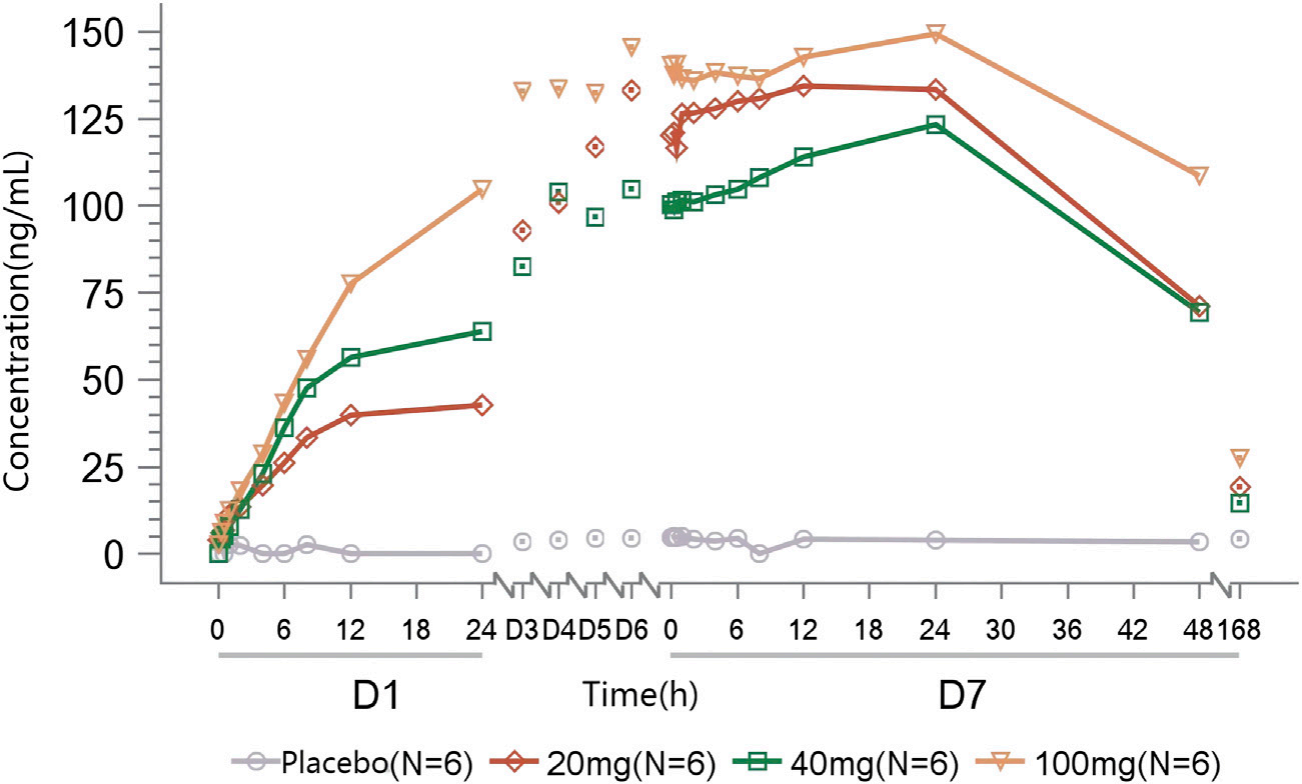
Mean methylamine concentration–time profile after multiple-dose administration.

A dose-dependent accumulation of plasma methylamine was observed on day 1 after the first dose of TT-01025-CL, with mean E_max_ ranging from 46.816 to 104.866 ng/mL. After multiple doses of TT-01025-CL, a further increase in plasma methylamine was observed on the last day of TT-01025-CL administration. Plasma methylamine appeared to plateau at doses of 20 mg and above, with mean E_max_ ranging from 124.142 to 156.070 ng/mL on day 7. No evident change in methylamine was observed in the placebo groups (Figure 6; Table 8).

**TABLE 8:** Summary of parameters of methylamine after multiple-dose administration.

	Placebo (*n* = 6)	20 mg (*n* = 6)	40 mg (*n* = 6)	100 mg (*n* = 6)
D1				
E_max_ (ng/mL)	1.752 ± 2.717	46.816 ± 10.016	69.899 ± 20.913	104.866 ± 29.801
T_Emax_(h)	0 (0∼8)	24.0 (12∼24)	24.0 (12∼24)	24.0 (24∼24)
AUEC_0–24h_ (h*ng/mL)	4.703 ± 9.142	798.335 ± 192.147	1,123.438 ± 240.662	1,602.930 ± 407.876
D7				
E_max_ (ng/mL)	6.329 ± 0.999	139.638 ± 23.639	124.142 ± 31.114	156.070 ± 41.355
T_Emax_(h)	6.500 (0.25∼48)	12.000 (1∼24)	24.000 (12∼24)	24.000 (0∼24)
AUEC_0–24h_ (h*ng/mL)	46.773 ± 50.536	3,159.315 ± 589.373	2,695.048 ± 807.601	3,409.268 ± 843.084
AUEC_0–48h_ (h*ng/mL)	84.240 ± 102.283	5,616.318 ± 983.937	5,004.718 ± 1,505.139	6,504.433 ± 1,606.539
AUEC_0–168h_ (h*ng/mL)	324.640 ± 354.935	11,035.448 ± 1,651.001	10,034.358 ± 3,617.607	14,665.223 ± 3,932.953

Data are presented as the mean (standard deviation), except for T_Emax_, which is shown as the median (min, max).

Abbreviations: E_max_, maximum concentration of methylamine; T_Emax_, time to reach maximum concentration of methylamine; AUEC_0–24h (48 h, 168 h),_ area under the concentration–time curve from time zero to 24 h (48 h, 168 h).

## 4 Discussion and conclusion

NASH has become the most common cause of chronic liver fibrosis and cirrhosis worldwide. Treatment of NASH is a major unmet need. So far, there is no approved therapy for the disease, with the exception of Zydus’ saroglitazar, which is only approved in India (Fraile et al., 2021). NASH is characterized by hepatic steatosis, inflammation, hepatocellular injury, and fibrosis. Over the last few years, potential new treatment options, primarily targeting metabolic aspects of NASH, including FGF21 mimics, thyroid hormone receptor β (THR-β), and GLP-1 receptor agonists, have demonstrated clinical benefits in NASH, particularly improvement in hepatic steatosis. Consistent with the complex and multifactorial pathogenesis of NASH, the hepatic inflammatory response is also a key pathophysiological mechanism in disease progression and the transition from NAFLD to NASH. Combining with drugs that directly target inflammation may help expedite strategies to reverse fibrosis and the progression of the disease. VAP-1 mediates the infiltration of immune cells into the liver, increases oxidative stress, promotes inflammation, and contributes to the progression of liver fibrosis (Smith and Vainio, 2007; Arndtz et al., 2017). Targeting VAP-1 may be a promising anti-inflammation approach, either alone or adjunct to other metabolic therapeutic strategies, for NASH (Lalor et al., 2007). Preclinical pharmacological studies have shown that TT-01025-CL represents a highly potent and selective irreversible inhibitor of VAP-1. Studies in an animal model of NASH have shown that TT-01025-CL significantly improves hepatocellular steatosis, lobular inflammation, and hepatic fibrosis. This study determined the safety, tolerability, and PK and PD profiles of TT-01025-CL in Chinese healthy subjects.

The safety results indicated that the good tolerability of TT-01025-CL was well-tolerated and safe in healthy adult subjects at all doses examined (single dose: 10–300 mg; multiple doses: 20–100 mg) with no severe AEs, serious TEAEs, or serious ADRs reported. All AEs that occurred in this study were mild or moderate. The incidence of AEs did not increase with the increase in drug dose, indicating that there was no dose-related concern on safety. The overall incidence of AEs and ADRs and the severity of AEs in the TT-01025-CL group were similar to those in the placebo group. In the TT-01025-CL group (*N* = 46), there were 27 TEAEs in 19 subjects, an incidence of 41.3%, and 16 ADRs in 13 subjects, an incidence of 28.3%. In the placebo group (*N* = 14), there were 6 TEAEs in 5 subjects, an incidence of 35.7%, and 4 ADRs in 3 subjects, an incidence of 21.4%.

TT-01025-CL demonstrated a potentially best-in-class safety profile in its class. Two other irreversible SSAO inhibitors, TERN-201 and BI-1467335, have been clinically evaluated in inflammatory diseases, including NASH, diabetic retinopathy, and Parkinson’s disease. TERN-201 is currently being tested for ameliorating chronic liver inflammation and fibrosis in NASH patients; the TEAEs mainly include constipation, taste disorders, headaches, and oral herpes (Jones et al., 2020). However, these adverse events were absent in this trial. The clinical development of BI-1467335 in NASH and retinopathy was suspended after a phase I study (Clinical Trials. gov NCT03927209) that identified a risk of drug interactions with the compound in NASH patients (inhibition of monoamine oxidase-B in the brain) (NASH, 2022). The drug interaction was mainly due to its high brain penetration and off-target effects on monoamine oxidase-B in the central nervous system. TT-01025-CL has shown low brain penetration (<2% of plasma concentration) (Supplementary Table S4) and excellent selectivity for monoamine oxidase-B (>4,700-fold selectivity) in preclinical studies (Supplementary Table S5), suggesting that TT-01025-CL is at a low risk of similar drug interactions. In the current phase 1 study with TT-01025-CL, no nervous system-related TEAEs were reported.

In preclinical PK studies, TT-01025-CL is metabolically stable in the *in vitro* liver microsomes of mice, rats, dogs, and humans and exists *in vivo* mainly in prototype form with only a small amount of acetylated metabolites in rat and dog plasma. Therefore, only the plasma concentration of the prototype form and related PK parameters were evaluated in this study. TT-01025-CL exhibited a greater than dose-proportional increase in exposure within the range of 10–100 mg in the SAD study. In the dosage range of 20–100 mg, the concentration of TT-01025-CL was in a steady state after continuous administration once daily for 5 days, and there was no apparent accumulation of plasma drug concentration, and the pharmacokinetic characteristics were linear.

In this study, two PD biomarkers, plasma SSAO-specific amine oxidase activity and methylamine accumulation, were investigated to understand the target engagement of TT-01025-CL after single or multiple dosing. VAP-1/SSAO exists *in vivo* in both a membrane-bound form and a circulating soluble form (sVAP-1), which is produced by proteolytic cleavage of its membrane-bound form. These two pools of VAP-1 protein may have distinct exposure/target occupancy relationships. *Ex vivo* measurement of plasma SSAO-specific amine oxidase activity can be used as an indicator of sVAP-1 activity. Since methylamine is an endogenous substrate of SSAO, the accumulation of plasma methylamine is expected to increase upon SSAO inhibition; thus, plasma methylamine accumulation is potentially a good biomarker of total SSAO activity *in vivo*, including soluble and membrane-bound forms of the protein. As expected with an irreversible inhibitor, prolonged PD effects were dissociated with the remaining TT-01025-CL concentration. The complete inhibition of plasma SSAO-specific amine oxidase was observed in all dose groups, irrespective of the number of doses. Plasma methylamine increased with dose after a single dose and was maintained until 168 h after administration, which was consistent with a slow turnover of SSAO protein. Further plasma methylamine accumulation was observed after multiple doses, reaching a plateau at doses of 20 mg and above. At a steady state, plasma methylamine was observed to plateau throughout the day, indicating complete and sustained VAP-1 target engagement across the dose range of 20–100 mg. Compared to plasma SSAO-specific amine oxidase activity, the drug exposure/plasma methylamine accumulation relationship shifted significantly toward higher doses, which may serve as a more accurate biomarker reflecting target engagement in the total VAP-1 repertoire *in vivo*. However, based on the current data, we cannot rule out the possibility that the reduction in sVAP-1 may also contribute to the loss of plasma SSAO activity. Unfortunately, we have not yet established a valid human sVAP-1 assay for clinical evaluation. The measurement of sVAP-1 shall be included in our future clinical studies, especially in targeted patient studies.

In this first study in healthy Chinese adults, TT-01025-CL showed rapid absorption and dose-dependent plasma exposure, with minimal accumulation in the dose range of 20–100 mg oral dose per day. The PD results demonstrated rapid and sustained target engagement in a dose-dependent manner. Overall, a once-daily dose of ≥20 mg can achieve complete SSAO inhibition at a steady state. Collectively, TT-01025-CL appears to be a potential best-in-class irreversible VAP-1 inhibitor to date, and its excellent safety, tolerability, and PK/PD warrant clinical evaluation of the full potential of this target in patients with NASH.

## Data Availability

The original contributions presented in the study are included in the article/Supplementary Material; further inquiries can be directed to the corresponding authors.

## Supplementary material

SUPPLEMENTARY FIGURE S1 Flow chart of the single-ascending dose study.

SUPPLEMENTARY FIGURE S2 Flow chart of the multiple-ascending dose study.

SUPPLEMENTARY FIGURE S3 C_max_, AUC, and dose relationship after single-dose administration (100 mg–300 mg).

SUPPLEMENTARY FIGURE S4 C_max_, AUC, and dose relationship after multiple-dose administration (20 mg–100 mg).
