# Supplementary figures and images for: The first selective VAP-1 inhibitor in China, TT-01025-CL: safety, tolerability, pharmacokinetics, and pharmacodynamics of single- and multiple-ascending doses
Source: Front Pharmacol. 2024 Apr 29;15:1327008. doi: 10.3389/fphar.2024.1327008 (PMC11089243; doi:10.3389/fphar.2024.1327008)

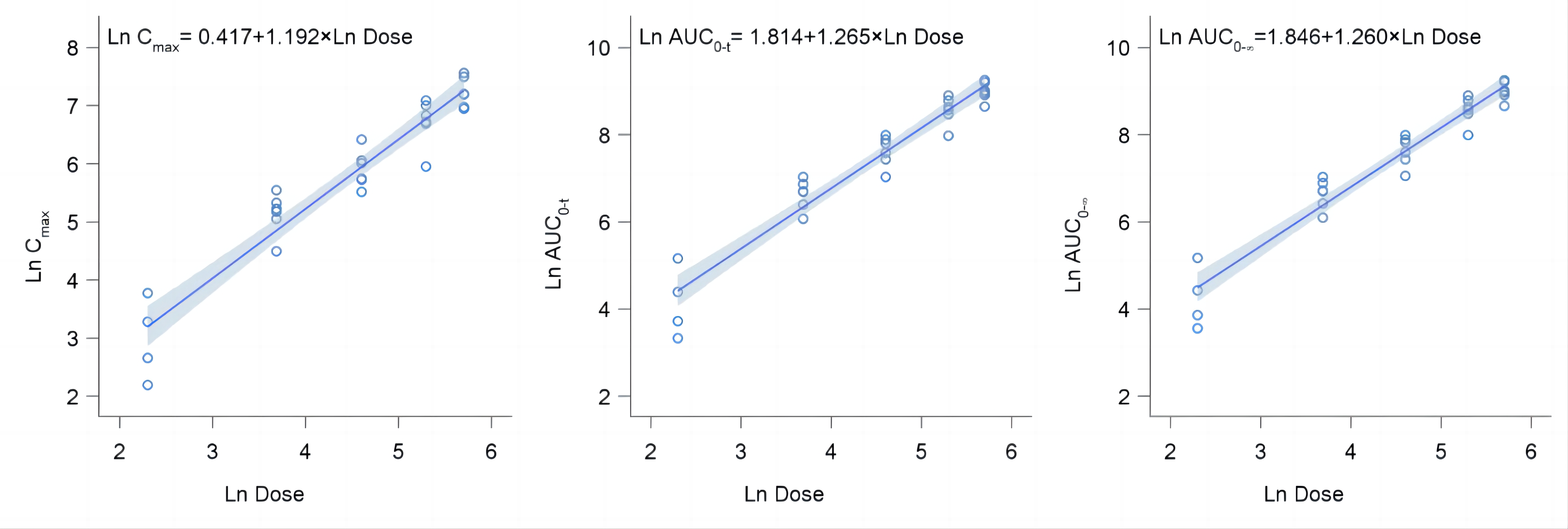

Supplement: Supplementary file 1 [file Image3.JPEG]

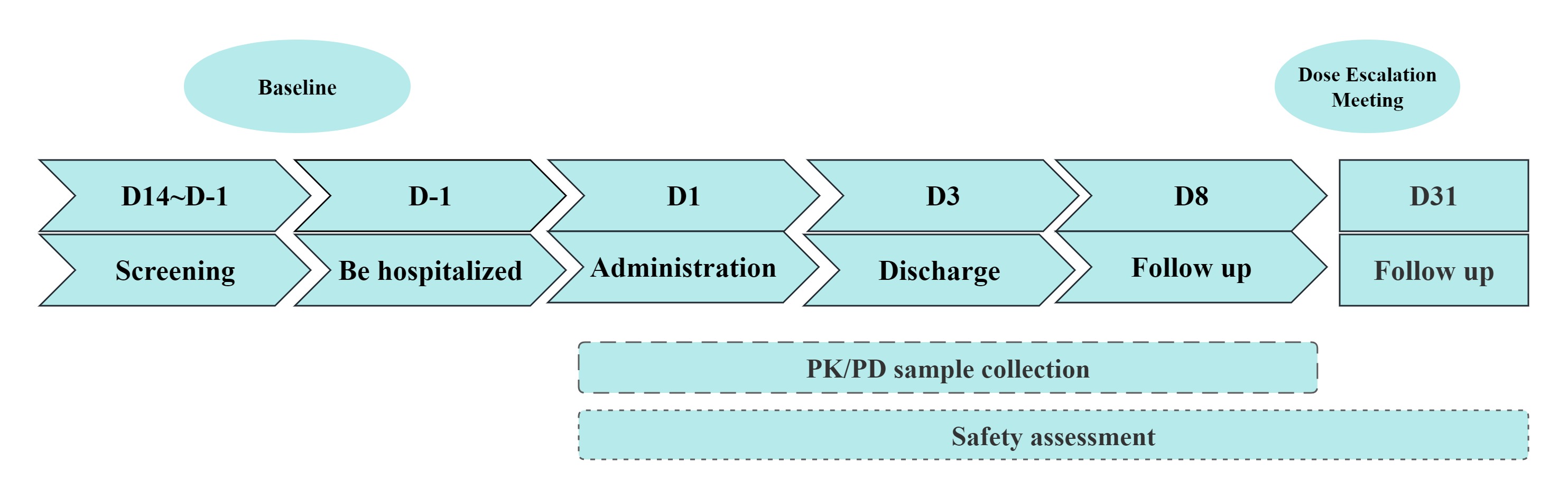

Supplement: Supplementary file 3 [file Image1.JPEG]

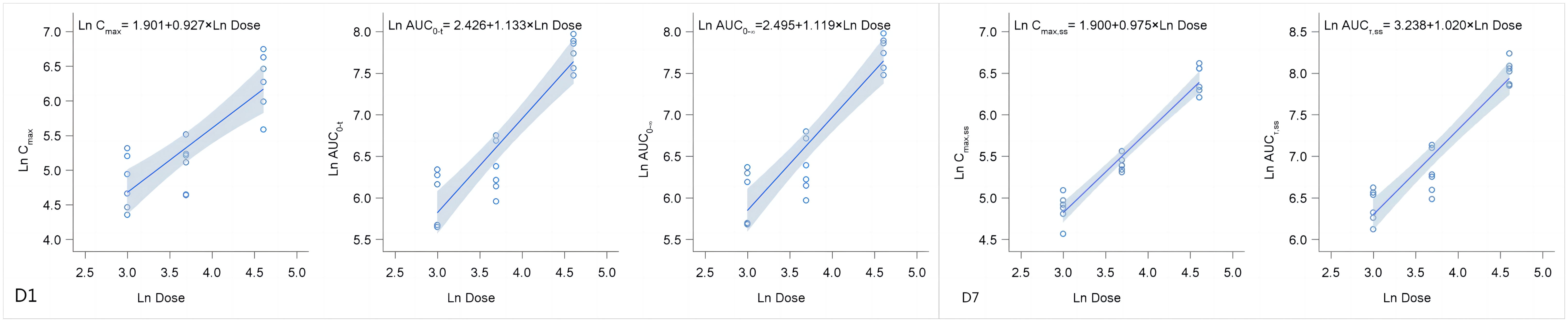

Supplement: Supplementary file 4 [file Image4.JPEG]

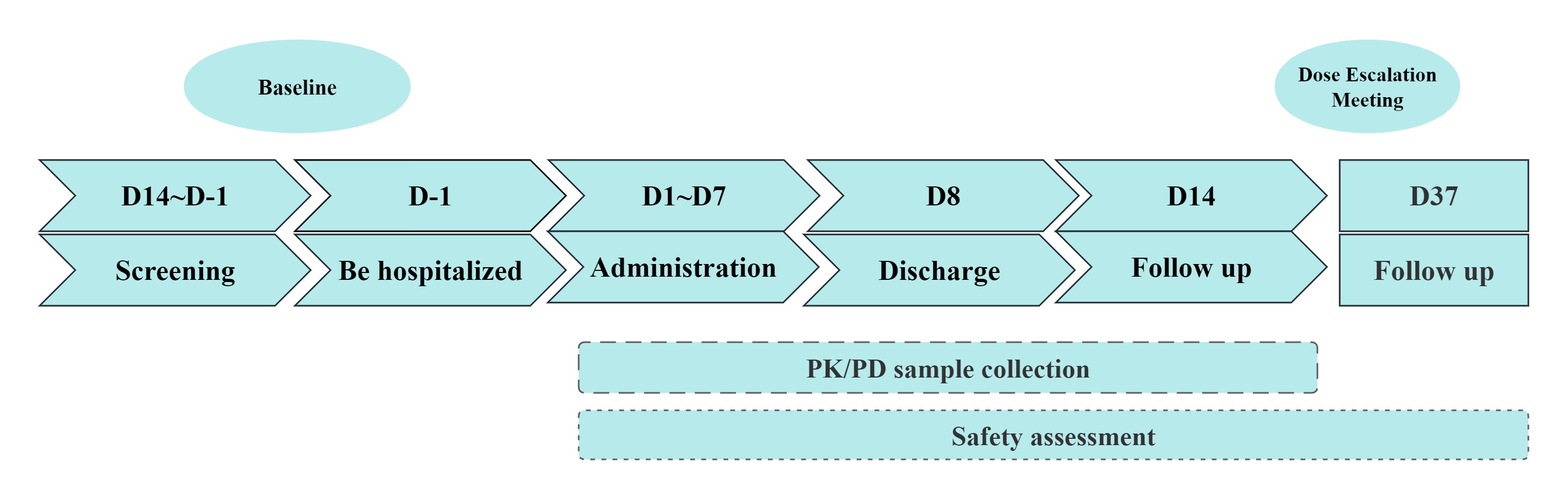

Supplement: Supplementary file 5 [file Image2.JPEG]
