# Supplementary material for: The first selective VAP-1 inhibitor in China, TT-01025-CL: safety, tolerability, pharmacokinetics, and pharmacodynamics of single- and multiple-ascending doses
Source: Front Pharmacol. 2024 Apr 29;15:1327008. doi: 10.3389/fphar.2024.1327008 (PMC11089243; doi:10.3389/fphar.2024.1327008)
Supplement: Supplementary file 2 [file Table1.DOCX]

**Supplementary Table S1** C_max_, AUC, and dose relationship after single dose administration

| Dose range | Parameters | Estimated value | SE | 95% CI |
| --- | --- | --- | --- | --- |
| 10mg~300mg | C_max_(N=28) |  |  |  |
|  | α | 0.445 | 0.318 | -0.209~1.100 |
|  | β | 1.195 | 0.069 | 1.053~1.338 |
|  | AUC_0-t_(N=28) |  |  |  |
|  | α | 1.233 | 0.330 | 0.555~1.912 |
|  | β | 1.384 | 0.072 | 1.236~1.531 |
|  | AUC_0-∞_(N=28) |  |  |  |
|  | α | 1.367 | 0.308 | 0.733~2.001 |
|  | β | 1.359 | 0.067 | 1.221~1.497 |
| 10mg~200mg | C_max_(N=22) |  |  |  |
|  | α | 0.399 | 0.393 | -0.421~1.219 |
|  | β | 1.209 | 0.092 | 1.016~1.401 |
|  | AUC_0-t_(N=22) |  |  |  |
|  | α | 1.065 | 0.405 | 0.220~1.911 |
|  | β | 1.432 | 0.095 | 1.234~1.631 |
|  | AUC_0-∞_(N=22) |  |  |  |
|  | α | 1.217 | 0.378 | 0.428~2.006 |
|  | β | 1.403 | 0.089 | 1.217~1.588 |
| 10mg~100mg | C_max_(N=16) |  |  |  |
|  | α | 0.182 | 0.505 | -0.901~1.266 |
|  | β | 1.277 | 0.133 | 0.991~1.562 |
|  | AUC_0-t_(N=16) |  |  |  |
|  | α | 0.828 | 0.547 | -0.344~2.000 |
|  | β | 1.507 | 0.144 | 1.198~1.816 |
|  | AUC_0-∞_(N=16) |  |  |  |
|  | α | 1.010 | 0.507 | -0.077~2.096 |
|  | β | 1.468 | 0.134 | 1.181~1.754 |
| 40mg~300mg | C_max_(N=24) |  |  |  |
|  | α | 1.253 | 0.433 | 0.356~2.150 |
|  | β | 1.036 | 0.089 | 0.852~1.219 |
|  | AUC_0-t_(N=24) |  |  |  |
|  | α | 2.198 | 0.395 | 1.379~3.018 |
|  | β | 1.193 | 0.081 | 1.025~1.360 |
|  | AUC_0-∞_(N=24) |  |  |  |
|  | α | 2.242 | 0.390 | 1.434~3.051 |
|  | β | 1.186 | 0.080 | 1.020~1.351 |
| 100mg~300mg | C_max_(N=18) |  |  |  |
|  | α | 0.417 | 0.875 | -1.437~2.271 |
|  | β | 1.192 | 0.167 | 0.837~1.547 |
|  | AUC_0-t_(N=18) |  |  |  |
|  | α | 1.814 | 0.805 | 0.108~3.520 |
|  | β | 1.265 | 0.154 | 0.938~1.591 |
|  | AUC_0-∞_(N=18) |  |  |  |
|  | α | 1.846 | 0.794 | 0.163~3.529 |
|  | β | 1.260 | 0.152 | 0.938~1.582 |

Using the power function model Ln (PK parameter) = Intercept(α) + β x Ln(Dose) + Error, the relationship between C_max_, AUC and dose was analysed.

Abbreviations: SE, standard erro; CI:confidence interval. C_max_, maximum concentration; AUC_0-t_, area under the concentration-time curve over from zero to the last quantifiable plasma concentration; AUC_0-∞_, area under the concentration-time curve from time zero extrapolated to infinity.

**Supplementary Table S2.** Linear regression analysis of three consecutive pre-administration based on trough concentrations

| Time | Dose | Estimated value | SE | 95% CI |
| --- | --- | --- | --- | --- |
| D5-D7 | 40mg(N=2) |  |  |  |
|  | Intercept | 0.729 | 0.992 | -2.024~3.482 |
|  | Slope | 0.046 | 0.164 | -0.409~0.500 |
|  | 100mg(N=6) |  |  |  |
|  | Intercept | 1.002 | 0.479 | -0.013~2.016 |
|  | Slope | 0.010 | 0.079 | -0.158~0.177 |

Steady state was judged to have been achieved by linear regression of pre-dose blood concentrations on three consecutive days (from D5) normalised by pre-dose blood concentrations on D7, with the 95% confidence interval for the slope of the regression containing 0.

Abbreviations: SE,standard error; CI ,confidence interval.

**Supplementary Table S3** C_max_, AUC, and dose relationship after multiple dose administration (20mg-100mg)

| Time | Parameters | Estimated value | SE | 95% CI |
| --- | --- | --- | --- | --- |
| D1 | C_max,ss_(N=6) |  |  |  |
|  | α | 1.901 | 0.585 | 0.662~3.141 |
|  | β | 0.927 | 0.153 | 0.603~1.251 |
|  | AUC_0-t,ss_(N=6) |  |  |  |
|  | α | 2.426 | 0.458 | 1.454~3.397 |
|  | β | 1.133 | 0.120 | 0.878~1.387 |
|  | AUC_0-∞,ss_(N=6) |  |  |  |
|  | α | 2.495 | 0.462 | 1.516~3.475 |
|  | β | 1.119 | 0.121 | 0.862~1.375 |
| D7 | C_max,ss_(N=6) |  |  |  |
|  | α | 1.900 | 0.219 | 1.436~2.365 |
|  | β | 0.975 | 0.057 | 0.853~1.097 |
|  | AUC_τ,ss_(N=6) |  |  |  |
|  | α | 3.238 | 0.340 | 2.517~3.960 |
|  | β | 1.020 | 0.089 | 0.832~1.209 |

Using the power function model Ln (PK parameter) = Intercept(α) +βx Ln(Dose) + Error, the relationship between C_max_, AUC and dose was analysed.

Abbreviations: SE, standard erro; CI: confidence interval. C_max,ss_, maximum concentration at steady state; AUC_0-t,ss_, area under the concentration-time curve from time zero to the last quantifiable concentration at steady state; AUC_0-∞,,ss_, area under the concentration-time curve from time zero extrapolated to infinity at steady state; AUC_τ,ss_ , area under the concentration-time curve at one dosing interval at steady state.

**Supplementary Table S4** The brain penetration data of TT-01025 and BI-1467335

| Chemicals | TT-01025-CL | BI 1467335 |
| --- | --- | --- |
| Brain/Plasma ratio | 0.0472 | 1.37 |

**Supplementary Table S5** Additional data on the selectivity of TT-01025-CL、BI-1467335 and TERN-201

| Targets | | SSAO | MAO-A | MAO-B | AOC1 | AOC2 | LOXL2 |
| --- | --- | --- | --- | --- | --- | --- | --- |
| TT-01025-CL | IC50/uM | 0.024 | >100 | >100 | 36 | 222 | 4.8 |
|  | Selectivity |  | >4000 | >4000 | 1500 | 9233 | 200 |
| BI 1467335 | IC50/uM | 0.005 | >100 | 2.7 |  |  |  |
|  | Selectivity |  | >20000 | 540 |  |  |  |
| Terns-201 | IC50/uM | 0.0065 | >50 | >50 |  |  |  |
|  | Selectivity |  | >7692 | >7692 |  |  |  |

Abbreviations: amine oxidase copper-containing; MAO: monoamine oxidase; LOXL:lysyl oxidase-like
